# Effects of an acute bout of dynamic stretching on biomechanical properties of the gastrocnemius muscle determined by shear wave elastography

**DOI:** 10.1371/journal.pone.0196724

**Published:** 2018-05-03

**Authors:** George M. Pamboris, Marika Noorkoiv, Vasilios Baltzopoulos, Hulya Gokalp, Robert Marzilger, Amir A. Mohagheghi

**Affiliations:** 1 Centre for Human Performance, Exercise and Rehabilitation, Brunel University London, Uxbridge, United Kingdom; 2 Institute of Environment, Health and Societies, Brunel University London, Uxbridge, United Kingdom; 3 Liverpool John Moores University, Research Institute for Sport and Exercise Sciences (RISES), Liverpool, United Kingdom; 4 Department of Training and Movement Sciences, Humboldt-Universität zu Berlin, Berlin, Germany; 5 Berlin School of Movement Sciences, Humboldt-Universität zu Berlin, Berlin, Germany; 6 University of Social Welfare and Rehabilitation Sciences, Tehran, Iran; Taipei Medical University, TAIWAN

## Abstract

**Aims:**

The aim of this study was to examine the acute effects of dynamic stretching (DS) exercise on passive ankle range of motion (RoM), resting localized muscle stiffness, as measured by shear wave speed (SWS) of medial gastrocnemius muscle, fascicle strain, and thickness.

**Methods/Results:**

Twenty-three participants performed a DS protocol. Before and after stretching, SWS was measured in the belly of the resting medial gastrocnemius muscle (MGM) using shear wave elastography. DS produced small improvements in maximum dorsiflexion (+1.5° ±1.5; mean difference ±90% confidence limits) and maximum plantarflexion (+2.3° ±1.8), a small decrease in fascicle strain (-2.6% ±4.4) and a small increase in SWS at neutral resting angle (+11.4% ±1.5). There was also a small increase in muscle thickness (+4.1mm ±2.0).

**Conclusions:**

Through the use of elastography, this is the first study to suggest that DS increases muscle stiffness, decreases fascicle strain and increases muscle thickness as a result of improved RoM. These results can be beneficial to coaches, exercise and clinical scientists when choosing DS as a muscle conditioning or rehabilitation intervention.

## Introduction

Stretching is generally used as part of a training program to increase flexibility [1,2], improve sports performance [3] and reduce the incidence of muscular injury [4–7]. Flexibility or maximum range of motion (RoM) available at a joint depends on both mechanical and neural factors [8–10]. Only a limited number of studies have examined the effects of DS on joint RoM, despite the increasing use of DS as a warm-up routine in sporting contexts [11–14]. Accordingly, it is not clear whether the expected increased flexibility in response to DS is based on the modification of the neural mechanisms and/or mechanical properties of the muscle-tendon unit (MTU).

Using a numerical optimization procedure [15,16], Herda et al. [12] demonstrated that the increase in maximum RoM after four 30s sets of DS, consisting of contraction of the agonist muscle groups (knee flexors) was due to a decrease in the overall MTU stiffness. Samukawa et al. [14] reported that DS involving contraction of the plantarflexors increased maximum ankle dorsiflexion RoM and was associated with a proximal displacement of the MTU of the medial gastrocnemius (MGM) whilst standing. Using B-mode ultrasonography and dynamometry, Mizuno and Umemura [15], reported that, DS involving contraction of the dorsiflexors, increased maximum dorsiflexion RoM of the ankle joint but had no effect on the passive mechanical properties of the MGM MTU. They attributed this change to the increased stretch tolerance. Therefore, contrasting scenarios about the outcome of DS on MTU stiffness have been reported, and contribution of the modification of the MTU stiffness to the expected and observed increased flexibility after stretching remains unclear.

From a logistics point of view, technical and financial factors limit application of the traditional methods to the assessment of MTU stiffness. The studies described above evaluated the proximo-distal displacement of the MTU and muscle stiffness either by measuring the relationship between the joint angle and passive torque developed as resistance to joint movement (i.e. torque-joint angle relationship); using a numerical optimization technique [16,17]; or by measuring the MTU displacement from a neutral to a fully stretched position using a combination of motion analysis, dynamometry and ultrasonography [9,18–20] (i.e. force-displacement relationship). However, passive torque measurements and MTU displacement are influenced by several *in series* and *in parallel* factors such as properties of the synergistic muscles, aponeurosis, tendon, joint capsules, ligaments, skin, and nerves around a joint [21,22]. Additionally, measurement of the MTU displacement is not possible in all muscles using current techniques. Moreover, stiffness of the gastrocnemius MTU evaluated by the numerical optimization method [16,17] has low reliability and cannot be used to evaluate a single muscle [23]. This method is also valid for multi-joint muscles and necessitates passive experiments at various different knee angles, which may limit its use in clinical practice and its applicability for other muscles [21]. Magnetic resonance elastography has been used to evaluate muscle stiffness represented by shear modulus in three dimensions [24–26]. However, the measurement can only be performed with the participant in a lying position inside a scanner, and the acquisition cost remains a limitation. To assess whether DS or other intervention reduces muscle stiffness, a direct quantitative and sensitive method that enables the assessment of a single muscle’s mechanical properties is required.

The problems faced with measuring single muscle stiffness can be overcome using shear wave elastography (SWE), an imaging technique which utilizes Acoustic Radiation Force Impulse (ARFI) imaging to generate shear waves (SW) that are then tracked with an ultrafast wave insonification technique [27], to measure shear wave speed (SWS) in a muscle. The speed of SW has been related to material properties since SW travels faster through stiffer tissues. When performed under well-controlled conditions, SWE has been shown to be a reliable technique for investigating alteration in muscle biomechanical properties [21,23,28–32]. Furthermore, SWE does not require external manual compression applied by the operator on a tissue, hence, has the advantage of providing more objective outcomes [33].

Research using elastography has been conducted to assess the effect of treatment and rehabilitation interventions on muscular structures of a localized area within soft tissues in real time *in vivo* [28,34,35]. Six studies applied this technique to investigate the immediate effect of static stretching (SS) (ranging 2–10 minutes in duration) on gastrocnemius muscle stiffness [36–41]. Five studies [36–40] reported that SS decreased muscle shear modulus/speed. In contrast, Nordez et al. [41] reported no decrease in medial gastrocnemius muscle belly stiffness after 10 min of SS, however, they applied SS at 80%-86% of the maximum RoM [41]. Inconsistencies between these studies could be due to differences in the employed stretching types. Due to different effect SS and DS have on performance [42] one may assume that the effect of DS on MTU stiffness and RoM might also be different. For example, SS involves taking a joint through its range to a position where the tension is felt, holding it for a period of 10-30s, and repeating the process two to four times [43], whereas DS is performed as a controlled movement through the active range of motion for each joint [44]. To the best of our knowledge, no previous study has examined the effects of DS on muscle belly stiffness *in vivo*.

The goal of this study was to evaluate the acute effects of DS on ankle joint flexibility, and contribution of the medial gastrocnemius (MGM) muscle belly stiffness to the expected increased flexibility. Accordingly, ankle joint RoM, SWS in the MGM, muscle fascicle strain and thickness were examined before and after the DS intervention.

## Materials and methods

### Participants

**Fig 1:**
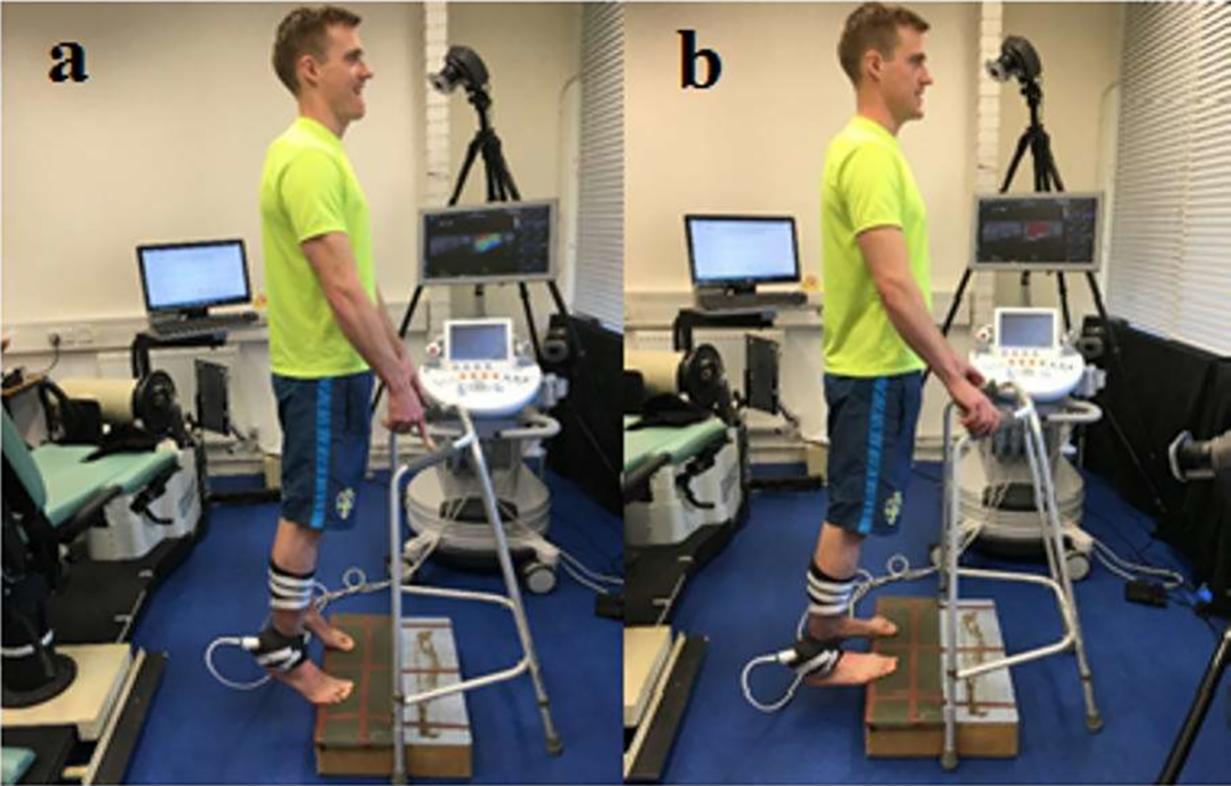
Start and finish position during the stretching protocol. (a) Standing erect on the step and (b) Position at full stretch.

Seventeen healthy men [age: 35.4±12.1 years; height: 1.78±0.06 m; body mass: 79.41±14.20kg] and six healthy women [age: 31.2±11.1 years; height: 1.61±0.05m; body mass: 60.50±7.04kg] volunteered to participate in this study. Participants were informed of the purpose of the study and methods used providing written consent. The experimental design of the study was approved by the Research Ethics Committee of the College of Life and Health Sciences at Brunel University London and was conducted in accordance with the Declaration of Helsinki. The participant portrayed in Fig 1 has given written informed consent (as outlined in PLOS consent form) to publish the pictures. The participants were included in this study if they were healthy and did not have a history of traumatic hip or knee injury in the dominant leg during the previous six months. They were instructed to refrain from vigorous physical activity for 48 hours before the testing sessions.

Based on the literature, the fluctuation in female steroid hormones during the menstrual cycle does not seem to have substantial influence on the mechanical properties of the human muscle and tendon in vivo [45], and the examination of any potential effects of the menstrual cycle was beyond the scope of the current study, women with a regular menstrual cycle lasting between 28 and 32 days were included and tested at a non-specific period. Additionally, no significant effect of sex has been reported on stretching-induced changes in MTU stiffness and RoM [46,47], thus both genders were included in the study.

### Experimental design

A crossover controlled study (single group, repeated measures experimental design) was used for this study. Outcome measures were assessed before and after the dynamic stretching protocol. Time and ankle positions were the independent variables with two (pre-stretching and post-stretching) and four (fully stretched position, fully shortened and neutral position, standing) conditions, respectively. Passive ankle RoM, SWS measured by shear wave elastography, and MGM architectural parameters (fascicle length, muscle thickness) were the dependent variables.

### Warm-up

A 5-minute standardized warm-up on a stationary cycle ergometer (Ergomedic 818E Monark, Stockholm) with a pedaling cadence of 60 rpm [48] was used. Participants were asked to adjust the seat to the correct height so that the knee was slightly flexed when the foot was parallel to the ground at the lowest pedal position [49]. This was implemented to avoid muscle injury and enhance the reliability of the measure [50]. All measurements were performed before DS (pre-DS) and after approximately 2 min of DS (post-DS). The two-minute interval was similar to the minimum period between warm-up and start of a game/training session as used by previous researchers “The two-minute interval was similar to the minimum period between warm-up and start of a game/training session as used by previous researchers who measured the effect of stretching on performance [44,51–54]. The participants were familiarized with the procedure and instructed to relax during the measurements.

### Dynamic stretching protocol

For performing DS, each participant wore unrestricted clothing and was asked to stand on a step. The participant started on the balls of both feet with the heels raised, lowering the heels in a controlled manner. The exercise was performed on the edge of the step to allow full dorsiflexion to be reached. The stretching exercise was performed at 100 beats/minute controlled by a metronome (MetroTimer 3.3.2, ONYX 3 Apps, Sofia, Bulgaria). Three sets of twenty repetitions were performed with a 5-second rest in between each set. Instructions were given to the participants to move into full plantarflexion (Fig 1A) and dorsiflexion (Fig 1B) during the protocol. In this way, we have standardized the muscle working length range during the DS (i.e. DS from shortest to longest possible muscle length), and kept the neuromuscular adaptations that are known to be length-specific (e.g. force, activation, potentiation) as consistent as possible in all participants.

### Instrumentation

#### Dynamometry testing of passive ankle angle/RoM

Participants adopted a supine position on an isokinetic dynamometer (Cybex NORM, New York, USA) with their knee in full extension and foot strapped to a foot plate to prevent the heel from lifting from the footplate. The rotational axes of the ankle joint and the dynamometer were visually aligned as closely as possible. Maximum dorsiflexion and plantarflexion passive RoM position were determined by manually moving the footplate. A slow angular velocity (<~5°/s) was used to ensure that the stretch did not elicit any reflex mediated muscle activity [55]. Participants were instructed to advise the researcher to stop at the point of discomfort. Throughout the movement, the participants were encouraged to relax and not resist the passive motion of the footplate. Ankle angle was measured at the maximum tolerated dorsiflexion and plantarflexion positions. The perpendicular position between the foot and the leg was considered the neutral position. (i.e. 0°). Angle data was recorded from the Cybex software.

#### Muscle shear wave elastic speed and fascicle strain

An Aixplorer ultrasound scanner (version 7.0; Supersonic Imagine, Aix-en-Provence, France) and a 50 mm linear array transducer (4–15 MHz, SL15-4, Vermon, Tours France) in supersonic shear imaging mode (musculoskeletal preset) were used to asses MGM shear elastic speed as previously described. Shear waves are generated in the tissue by focusing ultrasound pushing beams at different depths; then by using high-frame rate imaging (up to 20000 images/s), a movie of the shear wave propagating was recorded. B-mode images and SWS movies were acquired. The SWS was retrieved from a time of flight algorithm over the acquired movie [56]. Assuming a linear elastic behavior [27], the muscle shear elastic modulus (μ) was calculated as follows [57,58]:

$$\mu =\rho V_{S}^{2}$$

where *ρ* is the density of soft tissues (1,000 kg/m^3^) and V_s_ is the shear wave speed (m/s). Muscle is highly anisotropic [29], thus acquisitions were performed with the probe in the plane parallel to the muscle fibres and perpendicular to the skin; this position was determined when several fibres were continuously visible on the B-mode image. The preceding relationship is valid in tissues that are infinite (or large in extent), isotropic, homogenous, linear and elastic [59]. Since muscles do not have these characteristics this “stiffness” is reported in terms of shear wave speed (m/s) which requires few assumptions about the tissue geometry and mechanical coupling of tissue regions when measured at comparable joint angle or contraction state.

Simultaneous B-mode images were taken to assess the muscle architecture [27,60]. The ultrasound probe was placed longitudinally on the skin surface at 30% of the lower leg length measured distal to the lateral joint line of the knee [28,61–63] over the gastrocnemius muscle belly. The transducer was secured with a custom-made cast placed on the skin according to the orientation of the muscle fascicles and was securely bandaged to the leg with Cohesive Bandage (CURRAGH Veterinary Supplies, Culworth, Oxfordshire) to minimise undesired translation of the transducer. Water-soluble transmission gel (Henleys Medical Supplies Ltd., Hertfordshire, UK) was applied onto the contact surface to avoid excessive air gaps between the ultrasound probe and the dermal surface during the measurements.

**Fig 2:**
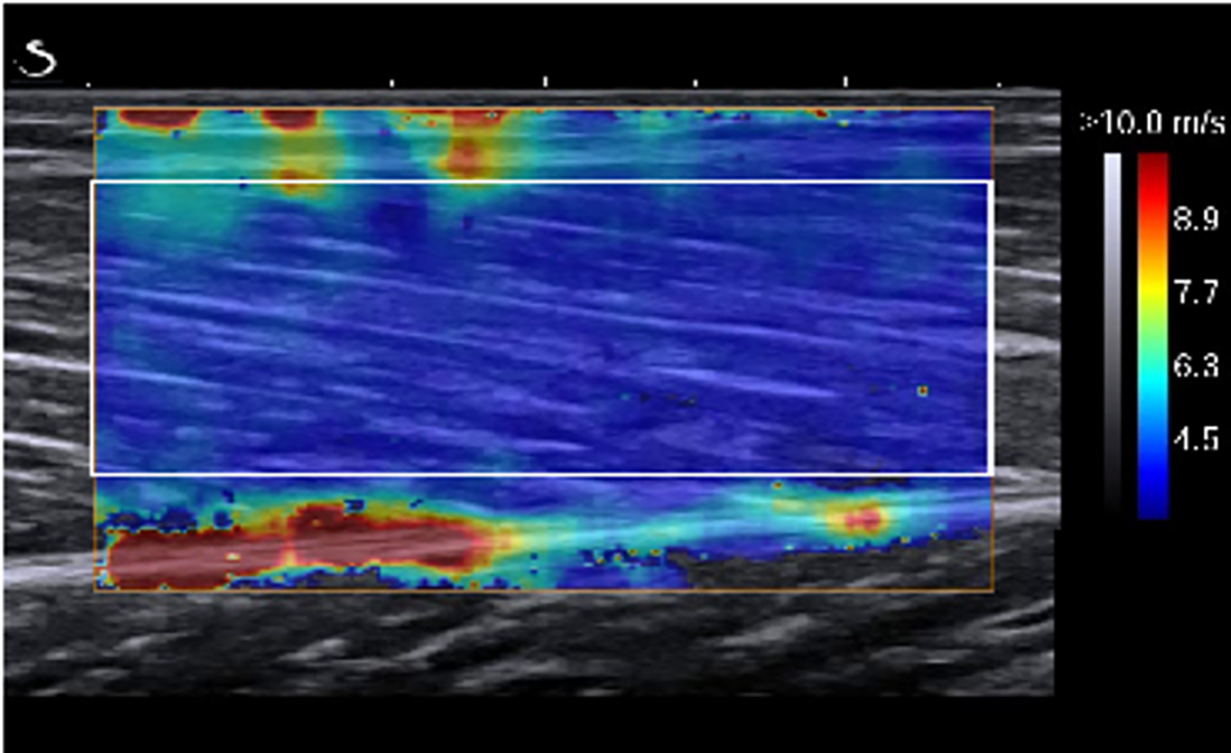
Shear wave speed measurement from the shear-wave ultrasound elastography image at the neutral ankle position. The rectangle represents the region of interest (ROI) between the superficial and deep aponeuroses, and the colored region represents the shear elasticity map with the scale to the right of the figure.

The maps of the SWS were collected at 1 Hz within a 1 x 1 cm square (Fig 2) and with a spatial resolution of 1 x 1 mm. A good intra-day reliability of MGM shear modulus/speed has been previously reported [21,23,31,64–67].

### Data analysis

#### Processing of ultrasound images

For the assessment of muscle stiffness, supersonic shear (SSI) images were exported from the Aixplorer scanner software (Q Box) (version 7.0; Supersonic Imagine, Aix-en-Provence, France) in DICOM format. A custom-written software developed in Matlab (MathWorks, Natick, USA), was used to manually select rectangular sub-regions between the superficial and deep aponeurosis using a region of interest (ROI) as large as possible, avoiding the inclusion of artifacts for the muscle. The ROI of the first image was then automatically tracked on the following images, and RGB value of each pixel was converted into a SWS value by using the color barcode embedded in the original DICOM image. All elastography movies were processed by the same operator. Good reliability of the SSI technique has been demonstrated previously [31]. The SWS was measured at three muscle lengths (relaxed, neutral and stretched) in prone and at a standing position. Participants were asked to relax during each recording that lasted approximately 10 seconds (i.e. ~244 frames). For each position, the 3rd up to the 8th second (i.e. a total of ~146 frames) shear elastic measurements of the ROI were computed as the mean to obtain a representative value.

For the assessment of architectural parameters, ultrasound images were digitized using a custom-written routine in Matlab (MathWorks, Inc.; Natick, MA) [68]. MGM architecture was assessed at neutral, end RoM at maximum plantarflexion and dorsiflexion positions, with the probe positioned approximately over the muscle belly. At this site changes in muscle architecture have been shown to be relatively uniform [60] in B-mode ultrasound images. The upper and lower aponeuroses were manually identified in the custom written Matlab script by setting reference points along the aponeuroses that were approximated by a linear least-square fitting. Visible features of multiple fascicles were digitized, and a representative reference fascicle was then calculated on the basis of the orientation of the digitized fascicle portions. The fascicle length was determined as the Euclidean distance between intersection points of the reference fascicle with the two aponeuroses. Muscle thickness was calculated as the distance between the two aponeuroses at the intersections points with the reference fascicle and averaged from these two values.

#### Passive muscle fascicle strain

Strain (ε) of the MGM muscle fascicles and tendon was defined as the percentage of the change in length to the resting length. Thus:

$$\varepsilon =\frac{l-l_{o}}{l_{o}}\times 100$$

where *l* is the final length and *l*_*o*_ is the original length of the tissue. Fascicle strain was defined as the change from neutral to the maximum plantarflexed or dorsiflexed positions.

### Statistical analysis

Descriptive statistics were reported as means and SDs. Data analysis was undertaken using a post-only crossover trial with adjustment for a predictor spreadsheet [69]. Differences between trials were expressed as percentages determined from log-transformed and subsequently back-transformed data, with 90% confidence intervals (CI) reported as estimates of uncertainty to quantify the magnitude of the difference between pre-intervention and post-intervention outcome performance measures [70]. This is suggested to be the appropriate method for quantifying changes in athletic performance [70]. Dependent variables were analyzed either as log-transformed data [SWS at Nneutral, maximum plantarflexion, maximum dorsiflexion and standing position, muscle thickness] or raw data [RoM, fascicle strain] according to Hopkins’s [71] definitions. In athletic performance research, it has been argued that it is not whether an effect exists but how big the effect is that matters and the use of the P-value alone provides no information about the direction or size of the effect or the range of feasible values [70]. The magnitude of the effect size was classified as trivial (<0.2), small (0.2–0.6), moderate (0.6–1.2) or large (2.0–4.0) and extremely large (>4.0) via standardized thresholds [70]. The threshold value for the smallest worthwhile change was set at 0.2 of the between-subject standard deviation. Non-clinical inference was based on the disposition of the 90% confidence interval for the mean difference to this smallest worthwhile effect; the probability (percent chances) that the true population difference between trials is substantial (beneficial/detrimental) or trivial was calculated as per the magnitude-based inference approach [72]. Where the 90% CI overlapped the thresholds for the smallest worthwhile change in both positive and negative sense, the true effect was classified as unclear. In the event that a clear interpretation was possible, these percent chances were qualified via probabilistic terms assigned using the following scale: <0.5%, most unlikely or almost certainly not; 0.5–5%, very unlikely; 5–25%, unlikely or probably not; 25–75%, possibly; 75–95%, likely or probably; 95–99.5%, very likely; and >99.5%, most likely or almost certainly [70].

The magnitudes of the relationships between fascicle strain and SWS, and ankle angle and SWS were interpreted using Pearson’s product moment correlation coefficient, which were converted into 90% confidence limits using a spreadsheet [73]. The magnitude of the correlation coefficient was interpreted using an adapted Cohen’s scale [74]: 0.00–0.10, trivial; 0.10–0.29, small; 0.30–0.49, moderate; 0.50–0.69, large; 0.7–0.89; very large, 0.90–1.00 almost perfect [70]. An inference about the true (large sample) value of a correlation was based on uncertainty in its magnitude [72]: if the 90% confidence limits overlapped small positive and negative values, the magnitude was deemed unclear; otherwise, the magnitude was deemed to be the observed magnitude. The confidence interval was derived via the Fisher z transformation [75]. Inferences about the correlation between SWS and ankle angle and SWS and muscle fascicle strain were made with respect to the smallest worthwhile correlation of 0.10 [76].

### Reliability of the measurements

The repeatability/reproducibility of the muscle shear wave speed was determined from the values obtained from the 3^rd^ up to the 8^th^ seconds (i.e. a total of ~146 frames) shear elastic measurements. To this end, the interclass correlation coefficient (ICC), typical error (TE) and coefficient of variation (CV) of SWS were calculated for the 3^rd^ to the 8^th^ seconds across the 4 ankle positions using a spreadsheet provided by Hopkins [77]. To derive the within-subject variation as a coefficient of variation (CV), data were log-transformed (100 x natural algorithm) before analysis [77]. To describe absolute reliability, TE of measurement in raw units was expressed as CV (%) using the Hopkins’s [77] spreadsheet. Coefficients of variation of ≤10%, 10–25%, and ≥25% were considered of good, moderate and poor reliability, respectively [78]. To interpret the magnitude of a CV representing typical differences or changes in performance times, we doubled the CV before assessing it on the above scale [79]. Absolute reliability was expressed as CV for direct comparison with relevant reliability studies, and TE as an indicator to aid practitioners to determine whether the observed SWS changes were true physiological responses or measurements errors [77,80]. For a description of relative reliability, ICCs were determined using the same spreadsheet, with ICCs >0.75, 0.40–0.75 and <0.40 being considered as good, moderate and poor reliability, respectively [81]. Uncertainty in all estimates was set at 90% confidence limits.

## Results

The before and after stretching values with mean differences, effect sizes and qualitative non- clinical inferences based on post-only crossover trial analysis are given in Table 1.

**Table 1:** Descriptive statistics and mean differences in the DS performance measures along with effect sizes and qualitative inferences.

Performance measure	Pre-stretching (mean± SD)	Post-stretching (mean± SD)	Mean difference (±90% C.I.)	Effect size (±90% C.I.)	Likelihood (%) of DS being beneficial/trivial/detrimental	Qualitative Inference
RoM at Maximum Plantarflexion (°)	45.78 ± 6.95	48.04 ± 6.36	+2.3 ±1.8	+0.31 ±0.25	78/22/0	Likely increase
RoM at Maximum Dorsiflexion (°)	17.59 ± 6.00	19.09 ± 6.11	+1.5 ±1.5	+0.24 ±0.24	61/39/0	Possibly increase
Total ankle ROM (°)	64.86 ± 13.71	68.73 ±12.37	+3.9 ±2.2	+0.27 ±0.15	78/22/0	Likely increase
SWS at Neutral (m/s)	3.81 ± 0.82	4.20 ± 0.89	+11.4 ±7.3	+0.48 ±0.29	94/6/0	Likely increase
SWS at Standing (m/s)	4.12 ± 0.79	4.70 ± 0.16	+16.0 ±8.1	+0.74 ±0.35	99/1/0	Very likely increase
SWS at Maximum Plantarflexion (m/s)	2.34 ± 0.42	2.45 ± 0.40	+4.9 ±6.0	+0.28 ±0.33	66/33/0	Possibly increase
SWS at Maximum Dorsiflexion (m/s)	7.66 ± 1.26	6.66 ± 1.08	-12.9 ±7.5	-0.78 ±0.49	0/2/97	Very likely decrease
Fascicle strain (%) [dorsiflexion]	16.20 ± 11.67	14.21 ± 8.08	-2.6 ±4.4	-0.21 ±0.37	3/44/52	Possibly decrease
Fascicle strain (%) [plantarflexion]	-38.52 ± 13.04	-35.78 ± 10.16	+2.9 ±4.2	+0.21 ±0.31	52/46/2	Possibly decrease
Thickness at Neutral (mm)	19.19 ± 3.41	19.79 ± 3.22	+4.1 ±2.0	+0.21 ±0.10	56/44/0	Possibly increase

Note. SWS at Neutral, Maximum Plantarflexion, Maximum Dorsiflexion, and Muscle Thickness are reported as log-transformed data. RoM, total RoM, and Fascicle Strain are reported as raw data. Only SWS was measured during standing.

### Passive ankle RoM

DS intervention resulted in a *likely* increase in passive ankle plantarflexion RoM (moderate effect), and a *possible* increase in passive ankle dorsiflexion (small effect). DS showed a likely increase in total ankle RoM (small effect) as shown in Table 1.

### SWS

The DS intervention resulted in a *likely* increase in SWS at neutral ankle joint position (moderate effect) and a *very likely* increase in standing position (very large effect). The DS intervention showed a *possible* increase in SWS at maximum plantarflexion position (small effect) and a *very likely* decrease in SWS at maximum dorsiflexion position (moderate effect) (Table 1).

### Muscle fascicle strain

**Fig 3:**
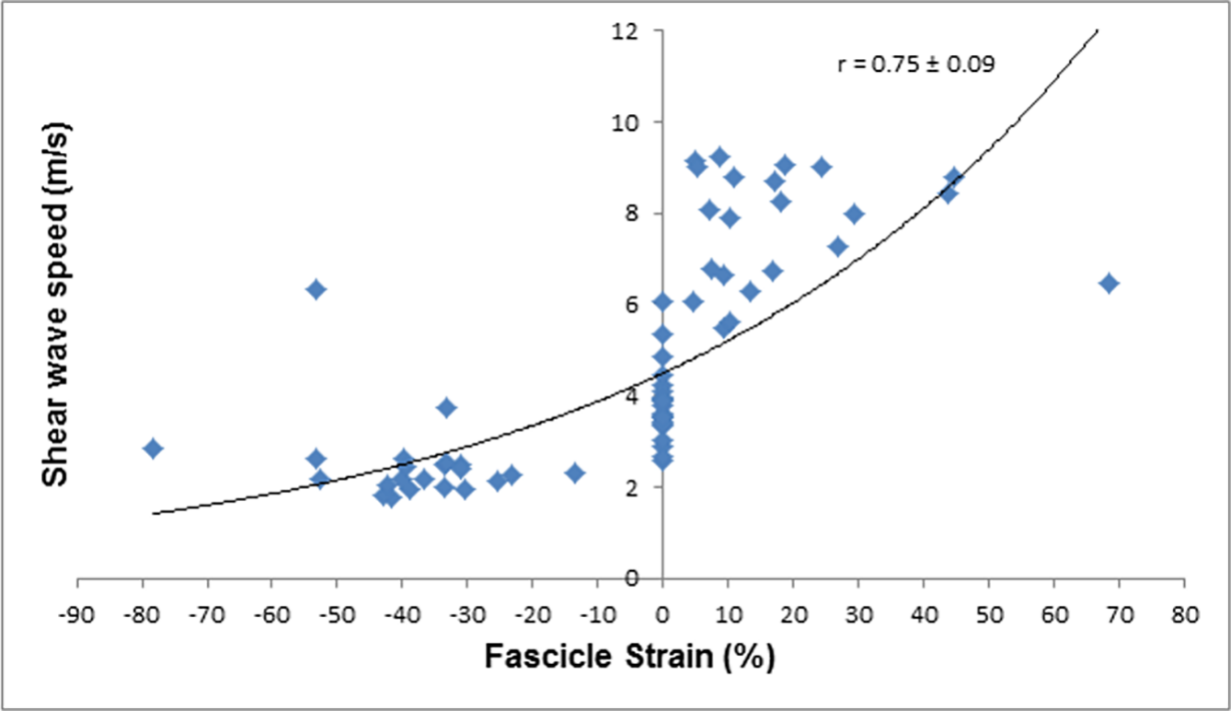
SWS of the MGM plotted against fascicle strain pre-DS.

**Fig 4:**
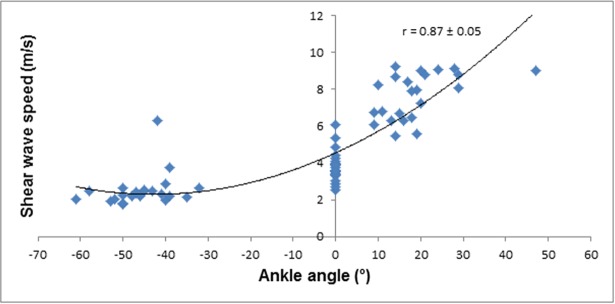
SWS of the MGM plotted against ankle angle pre-DS.

**Fig 5:**
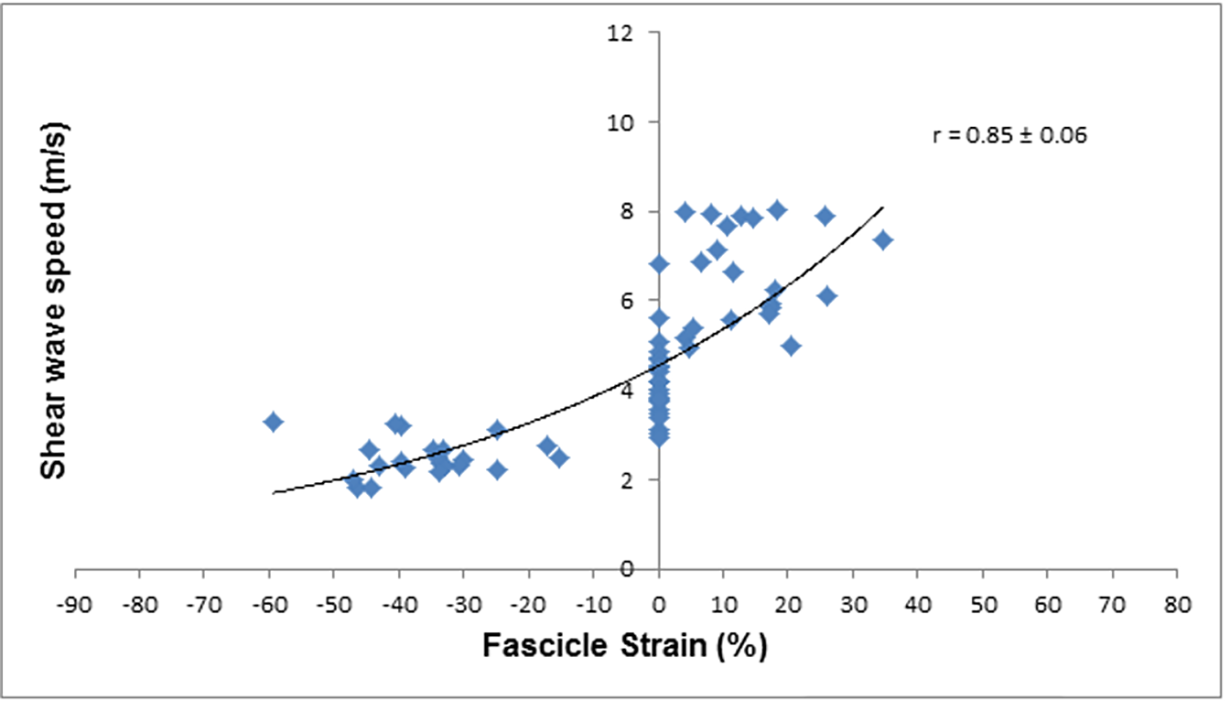
SWS of the MGM plotted against fascicle strain post-DS.

**Fig 6:**
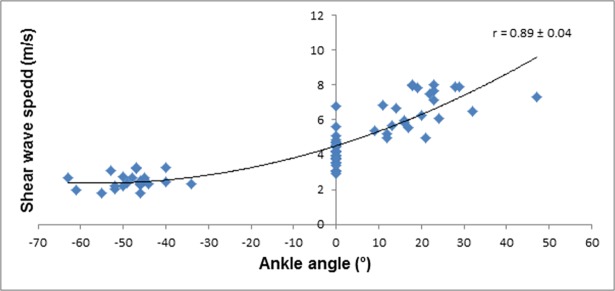
SWS of the MGM plotted against fascicle strain post-DS.

As expected, SWS was larger for the larger fascicle strains and at larger dorsiflexion angles prior to and post DS intervention. The relationships between SWS, fascicle strain, and ankle joint angle were characterized by a cubic fit with very large correlation coefficients (*r* = 0.75–0.89) and narrow 90% CI values (Figs 3–6).The DS intervention resulted in a *possible* decrease in muscle fascicle strain for both dorsiflexion and plantarflexion (small effect) (Table 1).

### Muscle thickness

As shown in Table 1, DS intervention resulted in a *possible* increase in muscle thickness at neutral ankle position (small effect).

### Repeatability of SWE measurements

The SWE results showed very good repeatability (relative and absolute) of this technique at all testing ankle positions and standing (Table 2). Neutral position was observed to have the highest relative and absolute repeatability.

**Table 2:** Repeatability results for SWS at 3 ankle positions and standing (n = 23).

SWS (m/s)				
Ankle position	Plantarflexion	Neutral	Dorsiflexion	Standing
Mean ± STD (m/s)	2.51 ± 0.93	3.81 ± 0.80	7.66 ± 1.3	4.19 ± 0.84
ICC (90% CI)	1.00	1.00	1.00	1.00
TE (m/s) (90% CI)	0.02	0.01	0.02	0.04
CV (%) (90% CI)	1.00	0.40	0.60	2.20

## Discussion

Joint angle, SWS, MGM muscle fascicle strain, and thickness were assessed *in vivo* before and after an acute DS protocol to examine the effect of this form of stretching on increasing flexibility. Our results showed that post DS intervention, SWS traveled faster in the MGM at the neutral ankle position, and therefore, DS has increased MGM tissue stiffness. This finding was associated with increased dorsiflexion angle, decreased muscle fascicle strain at the most dorsiflexed position and increased thickness. Moreover, SWS increased as ankle angle and fascicle strain increased which agreed with previous research that SWS for the MGM was dependent on ankle angle and fascicle strain [21,67,82].

The present study shows that SWE can be used for a reliable *in vivo* measurement of MG muscle stiffness. Our assessment protocol was designed to limit any differences caused by the operator. The high reproducibility of the measurements can be attributed to maintaining identical transducer orientation and location on the muscle throughout the measurements. In addition, tissue compression was minimised by fixating the probe on the muscle using a custom-made support and tape. This assured that the ultrasound probe position and pressure was the same during the pre and post DS elastography assessments and hence minimised the inter-operator error. The size of the elastography ROI from where the average SWS was automatically calculated using a custom written Matlab script, was identical between participants and also during the pre and post-DS. Therefore, this could not be a source of inter-observer error.

### Effect of dynamic stretching

As expected, an acute DS protocol increased dorsiflexion angle by 1.5° on average. Recently, an acute increase in dorsiflexion RoM after a DS protocol was reported to be between 3.1 to 7.3° [14,15]. Interestingly, the acute increase in plantarflexion was larger (2.3°). The *likely* increase in the SWS by ~10% immediately after stretching of the MGM at neutral ankle position, may imply that muscle stiffness increased due to DS. A number of previous studies investigated the immediate effect of SS only on muscle stiffness of the gastrocnemius [36–40] showing that SS decreased the muscle shear modulus, while Nordez et al. [41] reported no decrease in medial gastrocnemius muscle belly hardness after 10 min of SS. However, in this study, SS was performed at 80%-86% of the maximum RoM [41] and differences between the results might be due to the stretching technique employed.

Nevertheless, different mechanisms are proposed for the increased in RoM after SS compared to DS such as: (a) alteration in the mechanical properties of the muscle-tendon unit (MTU) (decrease in muscle-tendon stiffness [9,18,83,84] and passive resistive torque [19]), (b) decreases in muscle activation [85] or both [86]. The mechanisms that could explain the increase in RoM after DS are unclear.

Based on our results, we can speculate that the increased dorsiflexion RoM might be due to a decrease in stiffness of non-muscular structures such as such as synergistic muscles, tendon, skin, subcutaneous tissue, fascia, ligament, blood vessels, joint capsule, and cartilage. In other words, the opposite effects of DS on muscle might have occurred in the above s non-muscular structures in response to DS, where muscle stiffness increased but stiffness of the other structures decreased and resulted in an overall decrease in the joint stiffness and increase in RoM. “No alteration” in the MGM stiffness in addition to the reduced stiffness of the other structure stiffness could have led to the similar outcome (i.e. an overall decrease in the joint stiffness), however, our argument for the increase in MGM muscle stiffness after acute DS is based on the *likely* increase in the SWS at the neutral position. Additionally, the effect of an increase overall stretch tolerance cannot be disregarded.

Despite the increase in the MGM stiffness, SWS decreased at the most dorsiflexed position. The decrease in SWS in the most dorsiflexed position from pre to post-DS can be explained by the decrement in the fascicle strain which has been shown to be correlated to the SWS [21,67,82]. In other words, the reduced SWS at the most dorsiflexed position after DS protocol was because of the reduced fascicle strain and is not inconsistent with the reduction of the MGM stiffness although the contribution from changes in the pain tolerance [87–89] for the overall increase in flexibility cannot be ruled out.

Additionally, our results differ from those reported in previous studies in which DS was employed as a treatment intervention [12,15]. Mizuno and Umemura [15] found that DS (contraction of the antagonist to the target muscle group) does not change the mechanical properties of the muscle-tendon unit (stiffness) and attributed the change in RoM to enhanced stretch tolerance, whereas Herda et al. [12] reported that the increase in the RoM after four 30s sets of DS (contraction of the agonist muscle group to the target muscle group technique -knee flexors) was due to a decrease in the MTU stiffness. These results differ from the above where muscle stiffness was inferred from the passive torque-angle curve. These discrepancies may be explained by the fact that passive torque is influenced not only by muscular but also by non-muscular structures.

The connective tissue, and in particular the perimysium, is considered to be a major extracellular contributor to passive stiffness [90]. The ultrasound SWE scanner used in the present study can quantify localized tissue stiffness, and we exclude the outside connective tissues (epimysium, fascia, and aponeurosis) from the analysis of SWS data. Additionally, measurement of individual muscle properties is difficult to distinguish using dynamometry and measurement of the passive torque is affected by synergistic muscle activity, tissue composition and articular structures [21]. The method employed in the present study, i.e. SWE, allows quick and easy evaluation of the passive properties of individual muscles *in vivo* [31].

After the DS, there was a *possible* decrease in muscle fascicle strain at the most dorsiflexed position compared to the pre-stretching situation, which supports the notion that the increase in the overall elongation of the MTU may be attributed to the relatively increased contribution from the tendon. It should be emphasized that we did not measure tendon properties directly but considering that the muscle fascicle and the tendon are aligned in series, and both contribute to the MTU properties [9,19], a decrease in fascicle strain will imply an increase in the tendon strain. A future study should consider simultaneous measurements of the tendon properties to clarify the present results.

The decrease in muscle strain caused by the DS protocols may indicate microtrauma to the cytoskeleton-membrane and accompanying sarcomere disruption during eccentric exercise giving rise to Ca^2+^ levels in muscle fibers [76]. This, in turn, can trigger low-level activation and produces ‘contracture clots’, which increase passive musclee tension [77–79] by increasing cross-bridge formation and may lead to higher (resting) stiffness.

A recent study suggests that early increases in muscle SWS after exercise could reflect the pertuberation of calcium homeostasis induced by cytoskeletal alterations [91]. Although a direct relationship with the increase in SWS is not clearly established, a 28% increase in MGM SWS after eccentric exercise suggests that the MGM might have been damaged by this DS eccentric exercise protocol [92].

Several studies have reported increased muscle stiffness immediately after repeated eccentric muscle contractions [93,94]. Agten et al. [95] reported an immediate increase in SWS after eccentric exercise which was explained by extracellular muscle edema and increased blood flow due to the exercise rather than DOMS. Increased perfusion with exercise in our study might have produced ‘pseudohypertrophy’ of the muscle as indicated by increased thickness post DS. Metabolic factors may contribute to this, because adenosine triphosphate (ATP) is required to detach myosin from actin during cycles of muscle contraction [96]. With ATP loss during exercise, this detachment capacity decreases and the two proteins remain connected. Therefore, ATP loss may be another reason for the increased stiffness observed in SWS.

This work provides further evidence that SWS increase in MGM with increasing ankle angle and fascicle strain. These relationships are similar to the ones observed by other authors [21,67,82], however, there are some limitations to consider when interpreting the results of the study. We did not directly measure muscle activity throughout the passive dorsiflexion and plantarflexor movement to ensure that the muscles remained passive. However, the fact that the participants were instructed to stay relaxed throughout the testing procedures, and that we monitored fascicle length using B-mode ultrasonography and made sure that it stayed constant at the end RoM of passive ankle movements indicate that the muscle was quiet during the SWS passive measurements. In isotropic homogenous materials, SWS is directly related to the Young’s modulus [97], but this is not necessarily true in tissue which is transversely anisotropic [97], e.g. muscle. It is shown that SWS is related to the Young modulus in muscle [98] and provides a reliable measurement of the resting state muscle [31], but such relationship is unclear in passive and active measurements. Muscle is also anisotropic, viscoelastic and heterogeneous, which violates common assumptions needed to convert SWS to shear modulus and Young’s modulus. Therefore, we decided to report our values in SWS. Furthermore, SWS cannot differentiate which factors may contribute to the increased stiffness such as the extracellular matrix, intracellular proteins, or reflexively mediated activity [67]. A recent publication has shown that skin is a main contributor (among the tissues covering skeletal muscle) to the maintenance of muscle mechanical properties, contributing up to 50% to muscle shear modulus and that epimysium has no effect on muscle stiffness [99]. We can speculate that with any intervention we can change no more than around half of the factors influencing muscle stiffness. Another limitation is that SWS measurement was only performed at only one region of the muscle. Therefore, it is unclear whether the current findings can be generalized for the entire muscle region. Further research is needed to clarify this. Additionally, no study has investigated the acute effects of DS on SWS in elderly people and between genders. However, recent studies [100] have found no significant difference in MGM elasticity between men and women and between young and elderly women [101]. Recently Nakamura et al. [101] suggested that the effect of 5 min of SS on decreasing shear elastic modulus is similar between young and elderly women. Shear wave elastography assesses the muscle elasticity based on the shear wave propagation information within the defined ROI, which was placed on the muscle belly. Therefore, SWS can be measured without the confounding influences of the muscle volume or other nearby anatomical structures, such as synergistic muscles, tendon, skin, subcutaneous tissue, fascia, ligament, joint capsule, and cartilage compared to passive joint methods which is affected by muscle volume [102] and other structures within and around the joint (22).

Current results add to the body of knowledge by showing acute changes in single muscle properties in response to DS and support the notion that differential changes in the muscle and tendon mechanical properties, and hence function, can be achieved through different stretch training protocols. Our results have implications for dynamic muscle function in sporting and clinical contexts. For example, stiffer muscle combined with a compliant tendon is thought to transfer forces slower to the skeleton to initiate joint movements involving concentric contraction [103]. After DS, combined increased muscle stiffness and tendon compliance, might change muscle-tendon interaction during movement by shifting the optimum angle for force production, and/or contraction economy due to the altered elasticity of the tendon. More compliant tendons store more elastic energy than stiffer tendons under the same relative loading conditions [104,105]. When combined with a shorter and stiffer muscle during dynamic tasks, the larger tendon stretch and recoil increases the stretch-shortening cycle (SSC) rate and results in faster movements [103]. Additionally, a more compliant tendon is able to absorb more energy, during large SSC movements and accordingly, both the tendon and muscle will be less prone to injury.[103,106]. Future studies should measure the simultaneous effect on different structures around the joint, in addition to alteration in tendon properties directly.

## Conclusion

We have found that DS increases SWS in the resting MGM at the neutral ankle position. This suggests that the mechanical properties of the MGM may have altered, as evidenced by the greater muscle stiffness but also by a decreased muscle fascicle strain and increased muscle thickness. Results of the present study demonstrate the need for further exploration of the neuromuscular and functional adaptations to DS as a potential intervention that could be beneficial to athletes and in rehabilitation.
